# Supplementary material for: Untangling competition between epitaxial strain and growth stress through examination of variations in local oxidation
Source: Nat Commun. 2023 Jan 17;14:250. doi: 10.1038/s41467-022-35706-3 (PMC9842761; doi:10.1038/s41467-022-35706-3)
Supplement: Supplementary file 1 — Supplementary Information [file 41467_2022_35706_MOESM1_ESM.pdf]

Supplementary Information for Untangling competition between epitaxial strain and growth stress through examination of variations in local oxidation

Authors

Maria S. Yankova[1]*, Alistair Garner[1], Felicity Baxter[1], Samuel Armson[1], Christopher P. Race[1], Michael Preuss[1, 2]†, Philipp Frankel[1]

Affiliations

[1]Materials Performance Centre, Department of Materials, the University of Manchester, Manchester M13 9PL, UK
[2]Department of Materials Science & Engineering, Monash University, Clayton, 3800, Victoria, Australia
*maria.yankova@manchester.ac.uk
†michael.preuss@manchester.ac.uk

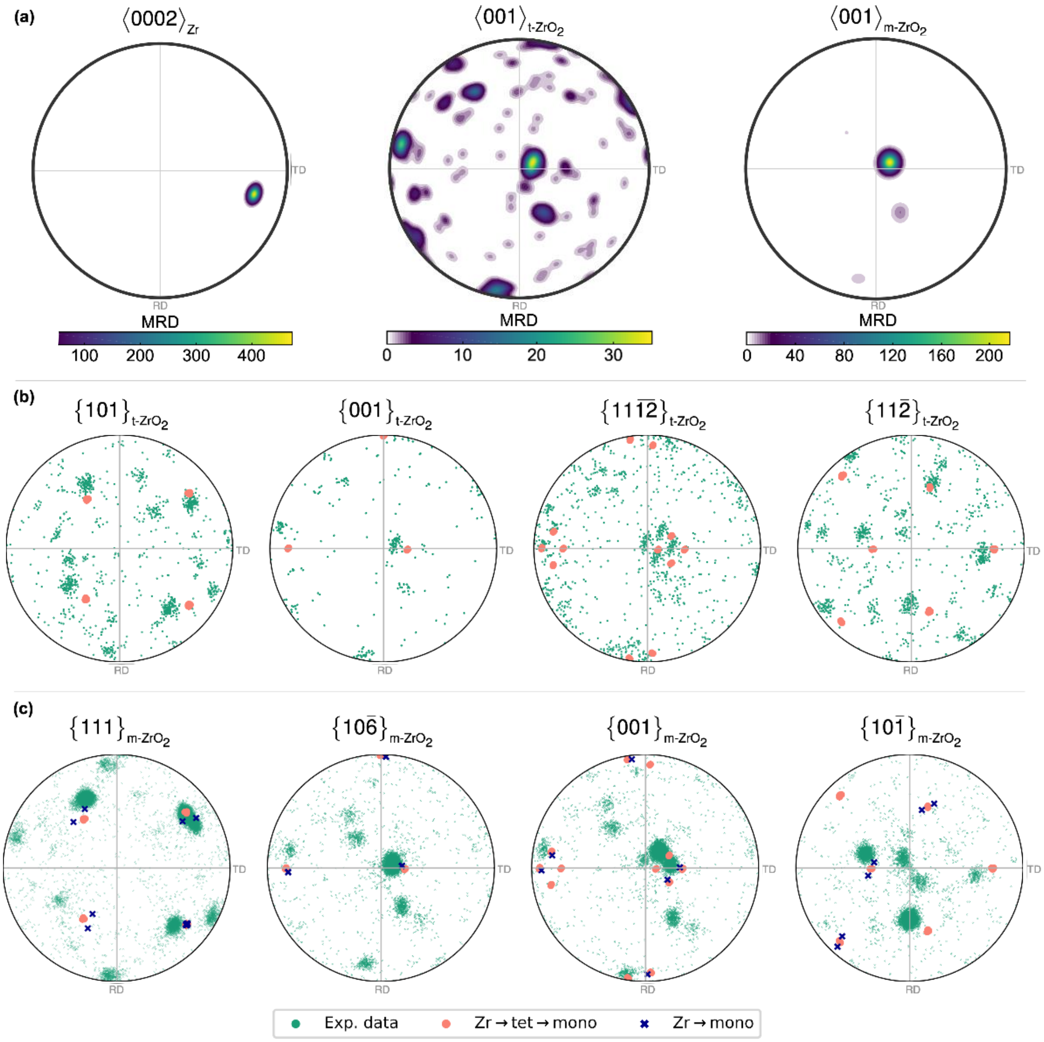

Supplementary Figure 1: EBSD crystallographic orientation data for oxide region 1' and metal grain 1'

(a) contoured pole figures for the ⟨0002⟩ pole in hexagonal Zr, and the ⟨001⟩ poles in tetragonal and monoclinic ZrO_2; (b) and (c) raw pole figures for equivalent poles {101}, {001}, {11$\bar{1}\bar{2}$} and {11$\bar{2}$} of

tetragonal and {111}, $\{10\bar{6}\}$, {001}, and $\{10\bar{1}\}$ of monoclinic ZrO_2; Denoted with pink disks and blue crosses are possible theoretical orientation relationships $\{111\}\langle10\bar{1}\rangle_{\mathrm{m-ZrO_2}}||\{101\}\langle11\bar{2}\rangle_{\mathrm{t-ZrO_2}}||\{0002\}\langle11\bar{2}0\rangle_{\mathrm{Zr}}$ and $\{111\}\langle10\bar{1}\rangle_{\mathrm{m-ZrO_2}}||\ \{0002\}\langle11\bar{2}0\rangle_{\mathrm{Zr}}$, respectively. All contoured pole figures are normalised to multiples of a random distribution (MRD) and oriented to be consistent with EBSD map in Figure 1 with TD || x axis, RD || y axis and ND || z axis.

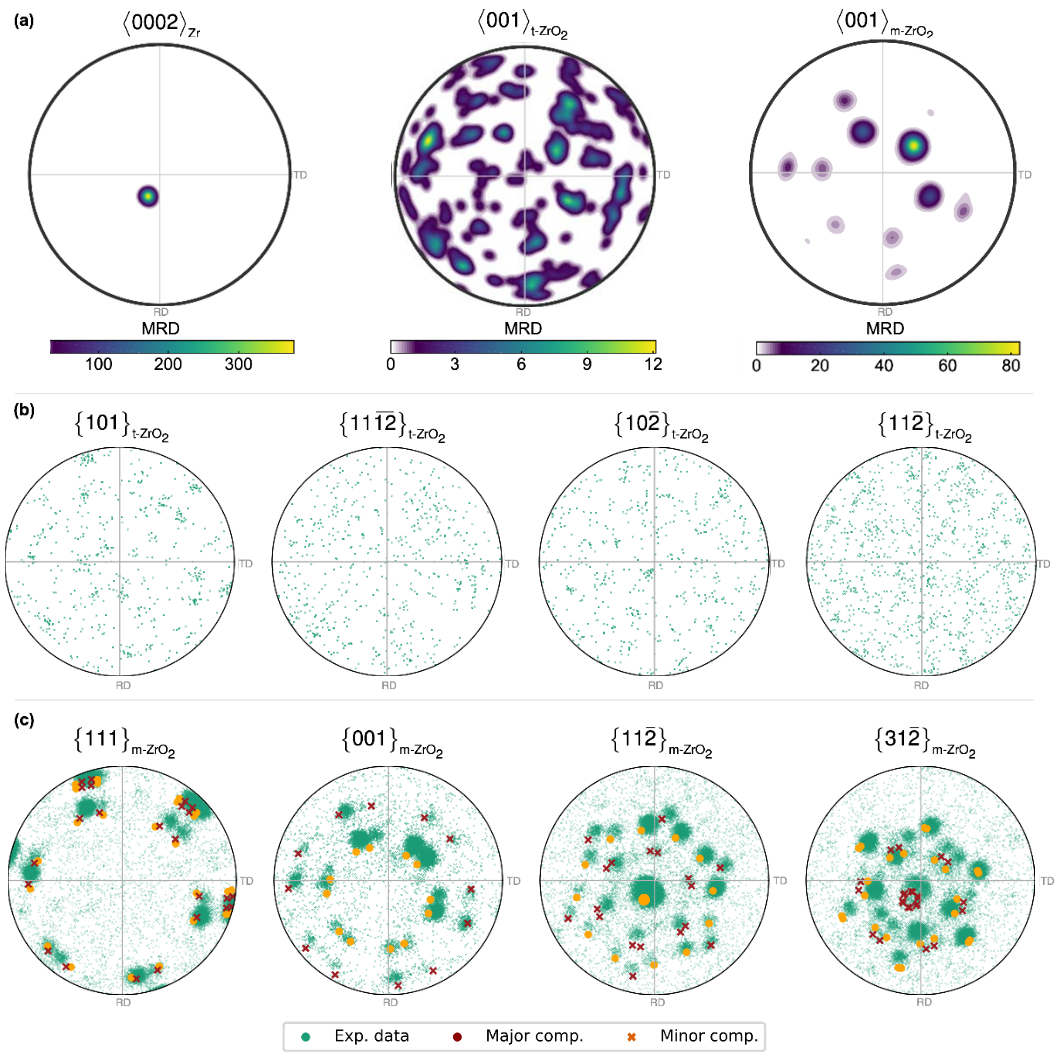

Supplementary Figure 2: EBSD crystallographic orientation data for oxide region 2' and metal grain 2'

(a) contoured pole figures for the $\langle0002\rangle$ pole in hexagonal Zr, and the $\langle001\rangle$ poles in tetragonal and monoclinic ZrO_2; (b) and (c) raw pole figures for equivalent poles {101}, $\{11\bar{1}\bar{2}\}$, $\{10\bar{2}\}$ and $\{12\bar{2}\}$ of tetragonal and {111}, {001}, $\{11\bar{2}\}$ and $\{31\bar{2}\}$ of monoclinic ZrO_2. Denoted with orange disks and red crosses are the major and minor theoretical orientation relationships $\{11\bar{2}\}\langle111\rangle_{\mathrm{m-ZrO_2}} \parallel \{0002\}\langle1\bar{1}00\rangle_{\mathrm{Zr}}$ and $\{31\bar{2}\}\langle111\rangle_{\mathrm{m-ZrO_2}} \parallel \{0002\}\langle1\bar{1}00\rangle_{\mathrm{Zr}}$. All contoured pole figured are normalised to multiples of a random distribution (MRD) and oriented to be consistent with EBSD map in Figure 1 with TD || x axis, RD || y axis and ND || z axis.
